# Comparative Analysis of Total Phenols, Total Flavonoids, Antioxidant Capacity, and Xanthine Oxidase Inhibition in Six Types of Tea

**DOI:** 10.3390/foods15101822

**Published:** 2026-05-21

**Authors:** Ke Wang, Jianpeng Zeng, Ying Chen, Yousheng Wang

**Affiliations:** 1 School of Light Industry Science and Engineering, Beijing Technology and Business University, Beijing 100048, China; wangke9509@163.com; 2 Institute of Modern Fermentation Engineering and Future Foods, Guangxi University, Nanning 530004, China; 19949968245@163.com; 3 Joint Research Institute of Future Foods and Comprehensive Health Industries, Guangxi University & Guangxi Zhongbo Nuocheng Biotechnology Co., Ltd., Nanning 530028, China; 4 College of Light Industry and Food Engineering, Guangxi University, Nanning 530004, China

**Keywords:** tea, antioxidant capacity, xanthine oxidase inhibition, principal component analysis, partial least square discriminant analysis

## Abstract

Tea (*Camellia sinensis* L.) is a traditional plant and consumed as a beverage or medicinal drink. *Camellia sinensis* teas exhibit in vitro xanthine oxidase (XOD) inhibitory activity. The total phenols, total flavonoids, antioxidant capacity, and inhibitory effect on the XOD activity of 101 tea extracts were systematically analyzed. The total phenol content of green tea was 158.58 ± 4.15 mg gallic acid equivalents/g, and the total flavonoid content was 122.24 ± 4.17 mg rutin equivalents/g. The total phenol (75.36 ± 9.86 mg gallic acid equivalents/g) and flavonoid (62.95 ± 6.76 mg rutin equivalents/g) contents of dark tea were two-fold lower than those of green tea (*p* < 0.05). The antioxidant capacity of green tea was 494.32 ± 12.41, 434.88 ± 13.20, 614.99 ± 21.87, and 257.81 ± 8.56 mg Trolox equivalents/g as 1,1-diphenyl-2-picrylhydrazyl (DPPH), ferric ion reducing antioxidant power (FRAP), 2,2′-azino-bis(3-ethylbenzothiazoline-6-sulfonic acid(ABTS), and total reducing capacity, respectively. The antioxidant capacity of the dark tea group was the lowest. Black tea showed the numerically highest average XODi activity, while no significant difference in XODi activity was detected among green, yellow, and black teas (*p* > 0.05). The XOD inhibitory effect was positively correlated with total phenol, total flavonoid, and antioxidant capacities, revealing the relationship among tea polyphenols, antioxidant capacity, and XOD inhibition rate. Principal component analysis (PCA) and partial least square discriminant analysis (PLS-DA) showed that green tea had a strong antioxidant capacity and inhibitory effect on XOD. These findings provide an in vitro screening reference for tea resources with antioxidant and xanthine oxidase inhibitory activities, and further studies on key component identification and in vivo validation are warranted.

## 1. Introduction

Globally, tea (*Camellia sinensis* L.) is the most consumed drink next to water, likely owing to its refreshing properties, pleasant taste and flavor, health benefits, and sociocultural characteristics. Tea has received increasing research interest lately due to its potential health benefits and application in food industries [1,2,3]. Several studies have been conducted to reveal the health benefits associated with consuming tea. Tea prevents several oxidative stress-related diseases and health conditions, including metabolic, inflammatory, and neurodegenerative diseases [4,5]. The preventive effects of tea against diseases are related to its phytochemical active compounds and antioxidant activities [6]. However, the mechanisms of action by which tea prevents and alleviates hyperuricemia (HUA), an oxidative stress and metabolic disease biomarker, remain limited.

Polyphenols, including phenolic acids and flavonoids, form a large proportion of the bioactive compounds of tea. Polyphenols are natural antioxidants and have scavenging activity on active free radicals and reactive oxygen species (ROS) in the living systems and human body [7,8]. Although the human body has antioxidant damage repair mechanisms, excessive ROS and free radicals induce serious damage, which may lead to a series of metabolic diseases, including type 2 diabetes, cardiovascular diseases (CVDs), non-alcoholic fatty liver diseases (NAFLDs), and HUA [9,10]. Moreover, ROS and free radicals in the form of superoxide ion (•O_2_^−^), singlet oxygen (^1^O_2_), hydroxyl radical (•OH), and hydrogen peroxide (H_2_O_2_) attack biological macromolecules and promote inflammatory and neurodegenerative diseases, DNA damage, and cancer [9,11]. Therefore, supplementing and replenishing the antioxidant potential of the body by consuming food rich in polyphenols is necessary. Accordingly, researching and screening effective natural antioxidants has become paramount.

Existing research indicates that agents with robust antioxidant potential produce hypouricemic effects [12]. Xanthine oxidase (XOD), a limiting enzyme in purine metabolism, catalyzes the oxidation of hypoxanthine and xanthine to form uric acid (UA), whereas peroxide radicals and hydrogen peroxides are formed, which raises the oxidative level in the human body [9,13]. As human tissues and cells lack the uricase enzyme, UA is constantly excreted via the kidneys in the urine. However, excessive UA production or inadequate excretion results in elevated serum UA, eventually causing HUA. Consequently, XOD activity may contribute to HUA and oxidative stress in humans. XOD products physiologically contribute to homeostasis; however, they exert pathological effects when present at high levels. Therefore, decreased XOD activity to maintain a balance between UA production and its excretion is considered beneficial to human health [14].

Tea made from Camellia sinensis leaves have a potential inhibitory effect on XOD (the limiting enzyme for UA metabolism). Although multiple types of tea are commercially available, the specific xanthine oxidase inhibitory (XODi) activity of each tea (and level of processing) has not yet been elucidated. Moreover, the total phenol and flavonoid contents, as well as antioxidant activities of different types of tea, are not well-explored, which may directly affect tea XODi activity. In addition, during processing, tea passes several unit operations, such as harvesting, fixation, fermentation, rolling, and drying, which exposes tea leaves to mechanical damage and chemical changes. The extent of processing and degree of fermentation induce changes in the bioactive compounds, color, flavor, and taste of tea, which renders different types of tea to be associated with different health benefits. Different types of tea with health benefits include green, white, yellow, oolong, black, and dark teas [15,16].

Here, we evaluated the antioxidant capacities and XODi activity of 101 samples of tea (green, white, yellow, oolong, black, and dark tea) using correlation analysis, multiple comparison, and principal component multivariate analysis approaches. The results of this study might be useful in guiding the selection of teas with high antioxidant capacities and potent XODi activity, and providing a reference for further in vivo and clinical studies on the UA-lowering functions of different types of tea.

## 2. Materials and Methods

### 2.1. Materials

Gallic acid (purity ≥ 98%) and rutin (purity ≥ 97.5%) (used as standards) were purchased from Yuanye Bio Co., Ltd. (Shanghai, China). Trolox [(±)-6-hydroxy-2,5,7,8-tetramethylchromate-2-carboxylic acid], 2,2′-azino-bis-(3-ethylbenzothiazole-6-sulphonic acid) diammonium salt (ABTS), 1,1-diphenyl-2-picryhydrazyl (DPPH), and 2,4,6-tri-2-pyridyl-s-triazine (TPTZ) were purchased from Sigma-Aldrich (Saint Louis, MO, USA). Folin–Ciocalteu reagents were purchased from Macklin Bio Co., Ltd. (Shanghai, China). Trichloroacetic acid (TCA), dibasic sodium phosphate (Na_2_HPO_4_), sodium dihydrogen phosphate (NaH_2_PO_4_), potassium persulfate (K_2_S_2_O_8_), ferric chloride (FeCl_3_·6H_2_O), potassium phosphate monobasic (KH_2_PO_4_), ethylenediaminetetraacetic acid disodium salt (EDTA), acetic acid, sodium acetate, sodium nitrite (NaNO_2_), aluminum nitrate (Al(NO_3_)_3_·9H_2_O), sodium hydroxide (NaOH), sodium carbonate (Na_2_CO_3_), and anhydrous ethanol were procured from Sinopharm Chemical Reagent Beijing Co., Ltd. (Beijing, China). Xanthine, allopurinol, and XOD were purchased from Sigma-Aldrich (Saint Louis, MO, USA). Other chemical reagents were of analytical grades. The standard compounds and solutions were either stored at −20 °C, 0 °C, or 4 °C until further use.

### 2.2. Tea and Tea Extract Preparation

Tea samples (*n* = 101) of green, white, yellow, oolong, black, and dark were obtained from various tea processing companies in China and Sri Lanka (Table S1). Tea samples were ground into a powder on a high-speed grinder (LD-Y300A, Shanghai Ding Shuai Electric Appliance Co., Ltd., Shanghai, China). The tea powder samples (1.5 g) were ultrasonically extracted three times with 15 mL 70% ethanol at 55 °C and 150 r/min for 30 min and filtered through filter paper. We selected 70% ethanol as the extraction solvent based on our preliminary experiments. This solvent system effectively extracts polar bioactive components in tea. The filtrate was combined to a set volume of 45 mL, centrifuged to remove the precipitation at 10,615× *g* for 10 min, and the supernatant was preserved.

### 2.3. Total Phenol Content Determination

The Folin phenol method was used to determine total phenol content in tea using a spectrophotometer (UV2450, Shimadzu, Kyoto, Japan), as previously described (with some modifications) [17]. Sample extract (0.05 mL), 0.85 mL water, 0.04 mL Folin reagent, and 0.06 mL Na_2_CO_3_ were added together. For the control group, water was used instead of the sample. Optical density was detected at 760 nm after 1 h dark treatment using a spectrophotometer (UV-2450-SHIMADZU). The same procedure was repeated for the standard gallic acid solutions to construct the standard gallic acid curve. The total phenol content was expressed as mg of gallic acid equivalents (GAEs) per gram of tea (mg GA equivalents/g tea).

### 2.4. Total Flavonoid Content Determination

Total flavonoids were determined according to the aluminum chloride (AlCl_3_) colorimetric method, as previously described (with minor modifications) [18]. Sample extract (50 μL) was added to 130 μL 5% NaNO_2_, 130 μL Al(NO_3_)_3_, and 690 μL NaOH. The optical density was measured at 510 nm on a UV-2450-SHIMADZU spectrophotometer following a 1 h reaction in the dark. Using water as the control, the antioxidant activity of the sample was determined according to the increase in absorbance value. The same procedure was repeated for the rutin standard solutions to construct the standard rutin curve. Total flavonoid content was expressed as mg of rutin equivalents per gram of tea (mg rutin equivalents/g tea).

### 2.5. Quantifying the Antioxidant Capacities of Tea

#### 2.5.1. DPPH Free Radical Scavenging Capacity

The radical scavenging capacity of the tea extracts was evaluated using a DPPH assay [17,19]. Tea extract (50 µL) was mixed with 150 µL of 70 mg/mL DPPH solution (diluted with anhydrous ethanol). For the control group, the sample was replaced with 50 µL water, and the DPPH working solution was replaced by absolute ethanol for the blank. The solution was shaken gently and kept away from light for 2 h at 25 °C. The detection wavelength for the decrease in absorbance was 520 nm when using a microplate reader (TECAN M1000 PRO, Männedorf, Switzerland) every 30 min. The standard curve was established using Trolox as the measurement standard. The DPPH free radical scavenging capacity was expressed as mg Trolox equivalents per gram of tea (mg Trolox equivalents/g tea).

#### 2.5.2. Ferric Reducing Antioxidant Power (FRAP)

A FRAP assay was conducted to evaluate the total antioxidant activity of tea extracts, as previously described [17,18]. Tea extract or Trolox (50 µL) were reacted with 135 µL TPTZ working solution and 15 μL acetic acid buffer (pH 3.6, 0.3 M) in microplate wells. The mixture optical density was measured at 593 nm, every 30 min, for 2 h using a microplate reader (TECAN M1000 PRO). The antioxidant activity was determined according to the increase in absorbance. Water was used as the negative control. FRAP antioxidant capacity was expressed as mg Trolox equivalents per gram of tea (mg Trolox equivalents/g tea).

#### 2.5.3. ABTS Radical Scavenging Activity

The antioxidant capacity of tea extracts, measured by ABTS radical scavenging, was determined as previously described [20]. Tea samples or water (50 µL)) were reacted with 150 µL ABTS working solution (A734 = 1 ± 0.02) away from light for 1 h. The optical density was measured at 734 nm wavelength every 10 min on a microplate reader (TECAN M1000 PRO). The ABTS radical scavenging capacity was expressed as mg of Trolox equivalents per gram of tea (mg Trolox equivalents/g tea).

#### 2.5.4. Total Reducing Capacity

Total reducing capacity was determined according to the previous method [17]. Briefly, 50 µL sample, 50 µL PBS (pH 6.6, 0.2 M), 50 µL potassium ferricyanide (1%), and 50 µL TCA were reacted and then incubated for 20 min at 50 °C. Thereafter, 60 µL of the reaction mixture was reacted with 120 µL water and 20 µL FeCl_3_. The optical density was determined at 700 nm every 20 min for 1 h using a microplate reader (TECAN M1000 PRO). Total reducing capacity was expressed as mg of Trolox equivalents per gram of tea (mg Trolox equivalents/g tea).

#### 2.5.5. XODi Activity Assay

The XODi activity assay was performed according to a previously published method, with some modifications [21]. The assay involved a reaction of 50 µL tea extract sample (final concentration is 1.4 mg/mL) or water (control group), 35 µL of phosphate buffer (pH 7.5), and 30 µL of 1.7 mU/mL XOD. The mixture was incubated at 30 °C for 15 min, followed by the addition of 60 µL xanthine (51 µM). The absorbance for uric acid formation was measured at 290 nm immediately and every 5 min for a total of 30 min on a microplate reader (TECAN M1000 PRO). XODi activity was expressed in a percentage calculated based on Equation (1). In this assay system, the IC50 value of allopurinol was 0.1206 μg/mL.

(1) XOD inhibitory rate (%) = [(A_0_ − A_1_)/A_0_] × 100

where ΔA_0_ = change in control absorbance and ΔA_1_ = change in sample reaction absorbance.

#### 2.5.6. Statistical Analysis

All data were obtained from three independent experimental repeats. Data were presented as mean ± standard error mean (SEM). Microsoft Excel 2019 was used for data organization, and GraphPad Prism 8.0 and SPSS (IBM statistics 25) were used for analysis of variance (ANOVA) and performing Duncan’s post hoc multiple comparison of means. Origin 2026 was used for creating heat maps and conducting Pearson’s correlation analysis. SIMCA 14.1 was used to perform multivariate principal component analysis (PCA) and partial least square discriminant analysis (PLS-DA). The statistical significance was considered at a 5% level of significance, and *p* < 0.05 was considered statistically significant.

## 3. Results

### 3.1. Total Phenol, Flavonoid, and Antioxidant Capacities of 101 Tea Samples

**Figure 1:**
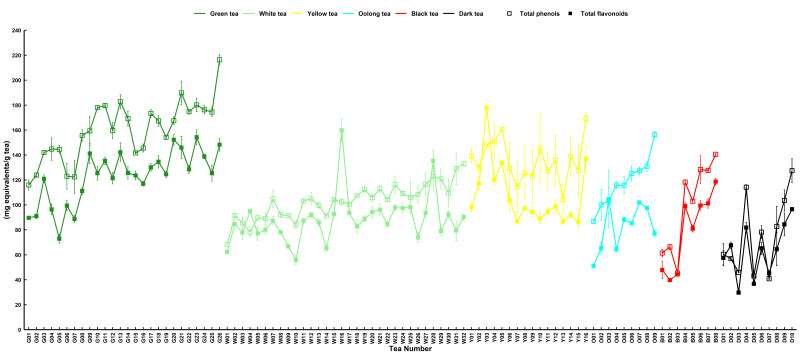
The total phenol and total flavonoid contents of 101 randomly selected tea samples. The standard error of the mean is shown by a vertical bar. Green tea (G); white tea (W); yellow tea (Y); oolong tea (O); black tea (B); and dark tea (D). DPPH, FRAP, ABTS, and total reducing capacity were used to evaluate the antioxidant capacity of different types of tea. Green tea (G13) had the highest DPPH scavenging capacity of 610.08 ± 4.34 mg Trolox equivalent/g tea, while dark tea D05 had the lowest DPPH scavenging capacity of 115.54 ± 0.83 mg Trolox equivalent/g tea (Figure 2). To further determine the contribution of different kinds of tea to antioxidants and other indicators, Duncan’s multiple comparative analysis was performed. The DPPH content of green and dark tea was the highest and lowest, 494.32 ± 12.41 mg and 256.15 ± 38.87 mg Trolox equivalent/g of tea, respectively (Table 1). DPPH capacity decreased in the following order: green > yellow > oolong > white > black > dark (Table 1). Duncan’s multiple comparisons indicated no significant differences in DPPH between green and yellow tea, white and oolong tea, and black and dark tea, whereas green, yellow, and black tea differed significantly, and dark tea reflected that the oxidation degree of tea also affected the DPPH content to some extent.

The total phenol and flavonoid contents differed significantly among tea samples (*p* < 0.05). Figure 1 shows that green tea G26 demonstrated the highest total phenol content at 189.89 ± 9.38 mg gallic acid equivalent/g tea, while dark tea D07 had the lowest content at 40.87 ± 0.63 mg gallic acid equivalent/g tea. The average polyphenol content for green tea samples was approximately 158.58 mg gallic acid equivalent/g tea, whereas yellow tea samples averaged 135.62 mg gallic acid equivalent/g tea, slightly lower than that of green tea (Table 1). Oolong, white, and black tea samples averaged at approximately 117.99, 102.98, and 98.89 mg gallic acid equivalent/g tea, respectively. Dark tea samples had an average polyphenol content of approximately 75.36 mg gallic acid equivalent/g tea. The total phenolic content of green tea was nearly double that of dark tea (Table 1). Overall, the order of total phenolic content was as follows: green > yellow > oolong > white > black > dark (Table 1). Green tea exhibited a high total flavonoid content of 122.24 ± 4.17 mg rutin equivalent/g tea, whereas dark tea showed a low content of 62.95 ± 6.76 mg rutin equivalent/g tea. Similarly, the order of total flavonoid content was as follows: green > yellow > white > oolong > black > dark tea (Table 1). In summary, green tea contained the highest levels of both total phenols and flavonoids, with yellow tea being marginally lower in both aspects. Dark tea had notably low levels of both total flavonoids and total phenols.

**Figure 2:**
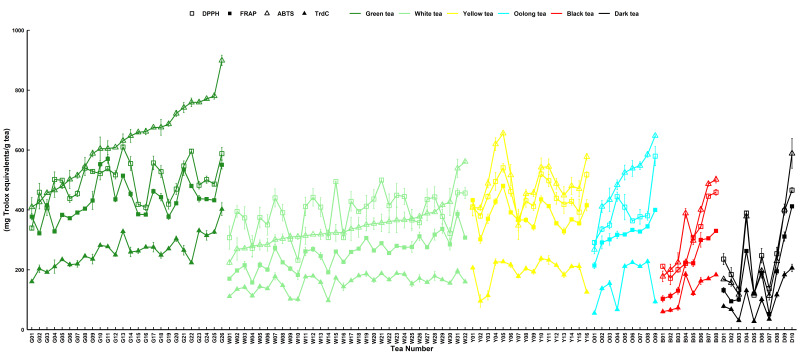
Antioxidant capacities of 101 randomly selected tea samples as presented by DPPH, FRAP, ABTS, and TrdC. The standard error of the mean is shown by a vertical bar. Green tea (G); white tea (W); yellow tea (Y); oolong tea (O); black tea (B); and dark tea (D).

Green tea (G11) had the highest FRAP of 570.68 ± 14.61 mg Trolox equivalent/g tea (Figure 2). The lowest FRAP content of dark tea D07 was 52.33 ± 3.34 mg Trolox equivalent/g tea (Figure 2). Dark tea also had the lowest overall value at 187.07 ± 35.47 mg Trolox equivalent/g tea, whereas yellow tea was slightly behind green tea FRAP at 384.38 ± 11.61 mg Trolox equivalent/g tea. The decreasing order of FRAP ability was as follows: green > yellow > oolong > white > black > dark tea (Table 1). Green tea differed significantly from other tea types in terms of FRAP value, which was attributed to their different contents (Table 1).

Regarding ABTS values, the mean antioxidant capacity of green tea was higher than that of black tea at 614.99 ± 21.87 and 247.04 ± 50.09 mg Trolox equivalent/g tea, respectively. The highest G26 content in green tea was 899.32 ± 17.61 mg Trolox equivalent/g tea, whereas the lowest content of D07 in dark tea was 112.89 ± 13.89 mg Trolox equivalent/g tea (Figure 2). The antioxidant capacity of ABTS was as follows: green > yellow > oolong > white > black > dark (Table 1). Duncan’s multiple test indicated significant differences between green and other tea types (Table 1). The differences and order of the antioxidant indexes were also consistent with the rules of other antioxidant indexes.

Green and dark tea had the highest and lowest total reducing power, respectively, at 257.81 ± 8.56 and 97.76 ± 19.76 mg Trolox equivalent/g, respectively, with the highest total reducing capacity of green tea G23 being 330.91 ± 5.78 mg Trolox equivalent/g (Figure 2). The decreasing capacity of total reducing power was as follows: green > yellow > white > oolong > black > dark (Table 1). In terms of total reducing power, the total reducing power of green tea samples was 257.81 ± 8.56 Trolox equivalent/g, with significant difference from other kinds of tea (Table 1).

### 3.2. Tea XODi Activity in 101 Tea Samples

**Figure 3:**
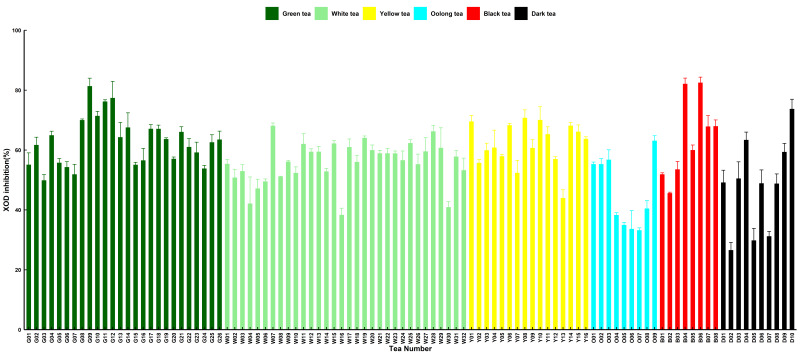
Xanthine oxidase inhibitory (XODi) activities of 101 selected tea samples. The standard error of the mean is shown by a vertical bar. Green tea (G); white tea (W); yellow tea (Y); oolong tea (O); black tea (B); and dark tea (D).

XODi activity was expressed as a percentage. Among the 101 samples, the sample with the highest XODi content was black tea sample B06, with an XODi value of 82.52%, and dark tea sample D02 had the lowest XODi value content of 26.57% (Figure 3). The average XODi activity of black tea was 63.95 ± 4.84%. The average XODi activity of green and yellow tea were also high at 63.21 ± 1.63% and 61.91 ± 1.84%, respectively (Table 1). Duncan’s multi-range comparison grouped green, yellow, and white tea together, with no significant difference between them, possibly because the fermentation process of the three teas was relatively similar (Table 1). In general, the order of XODi was as follows: black > green > yellow > white > dark > oolong (Table 1).

### 3.3. Correlation of Antioxidant Properties and XOD Inhibition Across Tea Types

**Figure 4:**
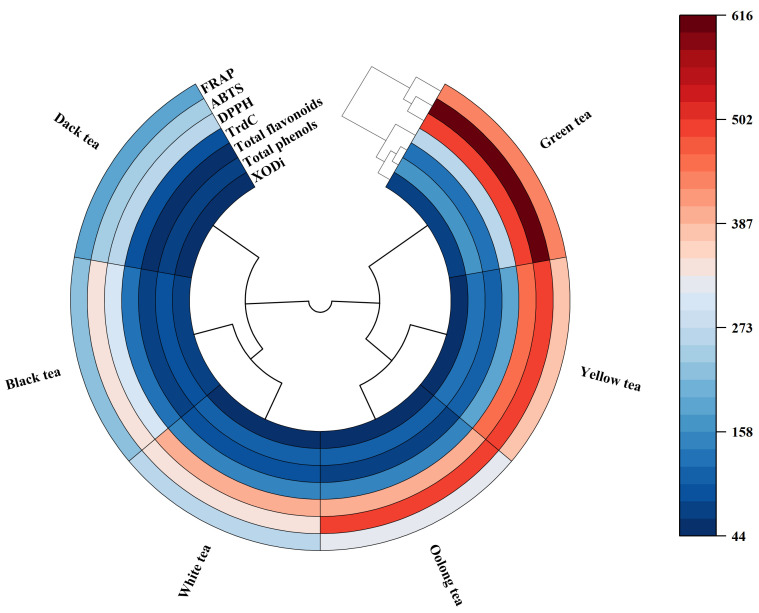
Heat map of antioxidant indexes of different kinds of tea. XODi: xanthine oxidase inhibitory; DPPH:1,1-diphenyl-2-picrylhydrazyl; FRAP: ferric ion reducing antioxidant power; ABTS: 2,2′-azino-bis(3-ethylbenzothiazoline-6-sulfonic acid; TrdC: total reducing capacity; TPC: total phenol content; TFC: total flavonoid content.

Figure 4 visually illustrates the correlations and differences among various tea species, including black, green, dark, yellow, white, and oolong tea, in relation to their antioxidant properties. This is represented through variations in color intensity and a tree-like branching structure. From the perspective of color depth, yellow and green tea notably exhibited strong antioxidant properties, and their colors are relatively similar. In contrast, dark tea ranked low across various antioxidant indices, which aligns with previous data analyses.

The phenolic compounds analyzed include XODi, DPPH, ABTS, and total phenols. The cluster dendrogram derived from the heat map indicates a certain clustering relationship among these antioxidant properties, suggesting correlations between them.

Overall analysis of the heat map reveals significant differences in antioxidant properties across different types of teas. These disparities may be attributed to multiple factors, such as processing techniques and varietal differences. These findings provide a scientific foundation for evaluating tea quality and conducting functional research.

**Figure 5:**
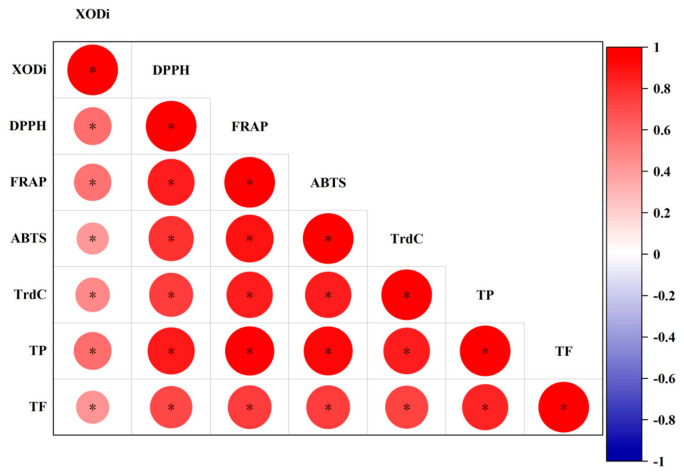
Correlation analysis matrix of tea indicators studied. The size of each circle indicates the strength of the correlation coefficient (^*^ *p *≤ 0.05). XODi: xanthine oxidase inhibitory; DPPH:1,1-diphenyl-2-picrylhydrazyl; FRAP: Ferric ion reducing antioxidant power; ABTS: 2,2′-azino-bis(3-ethylbenzothiazoline-6-sulfonic acid); TrdC: total reducing capacity; TPC: total phenol content; TFC: total flavonoid content.

In addition, Pearson’s correlation analysis showed that the antioxidant properties were all significantly positively correlated (*p* ≤ 0.05). The direction and degree of correlation can be judged by the red circle and the size of the circle (Figure 5). XODi and DPPH, FRAP, ABTS, TrdC, and total phenols and flavonoids were significantly positively correlated. Although its positive correlation with other antioxidant properties was slightly weaker, the increase in antioxidant properties could reflect the trend of XODi to some extent (Figure 2). Antioxidant capacity, total phenols, and total flavonoids in tea were significantly positively correlated with XOD inhibitory activity. Although the order of XODi content and antioxidant capacity of different tea species in the data are slightly different, the overall trend reflects that certain reactions occur.

### 3.4. PCA of Antioxidant Capacity, Total Phenols, Total Flavonoids, and XODi Activity of 101 Tea Samples

PCA represents the correction coefficient matrix of the original data set as the corrected eigenvalues and loading vectors. Using the PCA‑X model in SIMCA 14.1, sample scores and loadings were displayed within the 95% Hotelling’s T^2^ confidence ellipse.

**Figure 6:**
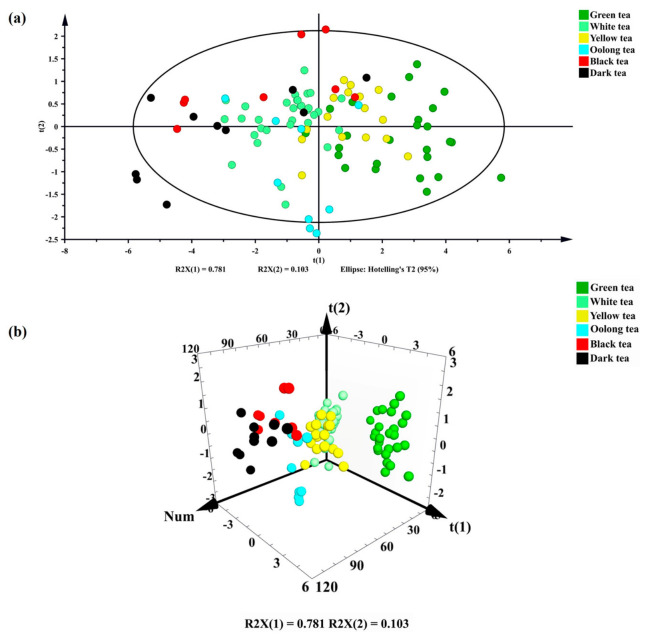
Multivariate analysis of 101 tea samples as antioxidant indices of total phenols, total flavonoids, and xanthine oxidase inhibitory activity. (**a**) Principal component analysis (PCA) of antioxidant indices for total phenols, total flavonoids, and xanthine oxidase inhibitory activity in 101 tea samples. (**b**) PCA-xyz-3d loading scatter. The type of tea group is indicated by the corresponding oval color: green tea: green dots; white tea: light-green dots; yellow tea: yellow dots; oolong tea: cyan dots; black tea: red dots; dark tea: dark dots.

Figure 6 shows the PCA plots of total phenols, total flavonoids, antioxidant capacity (DPPH, FRAP, ABTS, and total reducing capacity) and XODi activity of 101 tea samples. The PCA model explained 86.40% of the variance (R^2^X(1) = 0.781, R^2^X(2) = 0.103) and separated the different types of tea. The first principal component explained 78.1% of the variance, and the second principal component explained 10.3% of the variance. The cumulative explanation rate was approximately 88.4%, which indicated that the first two principal components could represent the data variation. The distribution of different tea samples in the two-dimensional space composed of the first two principal components (t(1) and t(2)) is shown in Figure 6a. Some types of tea have formed relatively notable aggregation classifications in the two-dimensional space, indicating certain differences between them and other distant aggregations in some aspects, such as antioxidant properties. The green, oolong, and yellow teas contributed positively to the PC1. The dark, black, and white teas contributed negatively to the PC1 component. However, black tea presented high loading scores on the PC2, which explained 10.3% variance of the studied indices. Green and dark teas were located on the positive- and negative-half axis of the horizontal axis and the difference is notable, which may reflect their differences in antioxidant properties and key components. The distribution of yellow and green tea is mainly located in the upper right part and the lower right part and there is a certain intersection, which may be because the light fermentation process of yellow tea is similar to the non-fermentation process of green tea. In general, most teas showed a certain clustering form.

In Figure 6b, the tea difference is further refined, and the overlap area of tea clusters becomes notable, especially for white and yellow tea. The reason may be that PC3 can reflect the subtle differences in chemical composition caused by different production processes of tea, such as oxidation and browning. The spatial location can also support the 2D figure to a certain extent, such as that green tea samples are mainly distributed in the positive-half axis of t(1) and the negative-half axis region of t(2), consistent with their high antioxidant content.

### 3.5. Antioxidant Capacity, Total Phenols, Total Flavonoids, and XODi Activity of 101 Tea Samples Determined Using PLS-DA

**Figure 7:**
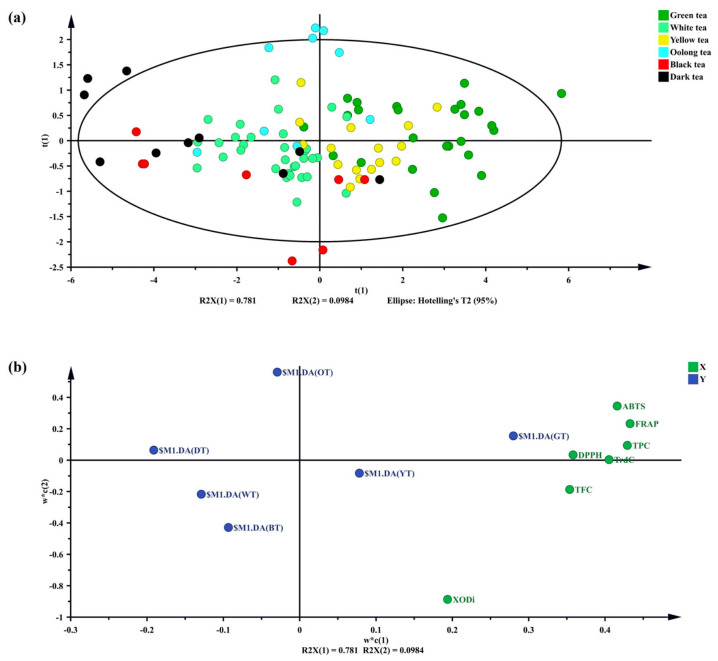
Partial least squares discriminant analysis (PLS-DA) plots of antioxidant indices, total phenols, total flavonoids, and XODi activities in 101 tea samples. (**a**) PLS-DA score plot. (**b**) PLS-DA loading diagram. The type of tea group is indicated by the corresponding oval color: green tea: green dots; white tea: light-green dots; yellow tea: yellow dots; oolong tea: cyan dots; black tea: red dots; dark tea: dark dots. XODi: xanthine oxidase inhibitory; DPPH: 1,1-diphenyl-2-picrylhydrazyl; FRAP: ferric ion reducing antioxidant power; ABTS: 2,2′-azino-bis(3-ethylbenzothiazoline-6-sulfonic acid; TrdC: total reducing capacity; TPC: total phenol content; TFC: total flavonoid content.

PLS-DA is a multivariate statistical method for classification and variable selection which is widely used in metabolomics, transcriptomics, and other fields. Latent variables were constructed to distinguish between different classes by maximizing the covariance between independent and dependent variables. Compared with PCA, PLS-DA can clearly associate classification targets with feature variables, which is suitable for the pattern recognition of high-dimensional and collinear data. In the study of tea species and antioxidant properties, this model can screen key metabolites and analyze their quantitative relationship with antioxidant activities (such as DPPH), optimize the classification accuracy, and provide a quantitative basis for the study of process–ingredient–function correlation. Cross-validation and VIP value should be combined to evaluate the robustness of the model and the importance of variables. The clear separation of green, yellow, oolong, black, white, and dark tea is shown in Figure 7a through PLS-DA scores. Figure 7b is the PLS-DA loading plot of total phenols, total flavonoids, antioxidant capacity (DPPH, FRAP, and ABTS), and XODi activity of 101 tea samples. The PLS-DA model explained 87.69% of the variance (R^2^X(1) = 0.781, R^2^X(2) = 0.0984) and separated different types of tea. The first principal component explained 78.1% of the variance, and the second principal component explained 9.84% of the variance. The cumulative explanation rate was approximately 87.94%, which indicated that the first two principal components could reflect the variation in the data to a good extent.

Figure 7a illustrates the total phenols, total flavonoids, antioxidant capacity (DPPH, FRAP, and ABTS), and XODi activity of 101 tea samples. Moreover, the notable separation of green, yellow, oolong, white, dark, and black tea is evident, which is echoed by the PCA score chart. In Figure 7b, green and yellow tea are located in the positive-half axis for the first PLS component (w*c(1)), which indicates that they have a high weight on this component and have an influence on the model. Simultaneously, the variables close to X are various antioxidant indicators such as total phenols, total flavonoids, XOD, FRAP, and ABTS. Consequently, green tea has high content and activity in these antioxidant indicators. This positional distribution may be related to the non-fermentation and micro-fermentation processes of green and yellow tea, resulting in the retention of relatively rich antioxidant components. Dark tea is a kind of post-fermented tea, which includes a special Wodui fermentation step. During fermentation, microorganisms metabolize compounds which form the unique aroma and mellow taste of dark tea. The chemical reaction produced by the complex interaction of microorganisms may lead to decreased antioxidant index content; therefore, dark tea falls in the negative-half axis of the first PLS component. In general, most tea leaves could be separated from each other. In this PLS-DA plot, green tea samples scored higher for the first PLS component (w*c(1)) and were close to antioxidant indicators such as FRAP, DPPH, ABTS, and XOD (Figure 7b). Accordingly, green tea has a high content or activity for these antioxidant indices, and likely has strong antioxidant activity. This distribution may be related to the processing of green tea, which is unfermented and retains a high content of tea polyphenols and catechins, which have strong antioxidant properties.

## 4. Discussion

Here, we report the antioxidant capacity and XODi activity of 101 teas, including green, white, oolong, yellow, black, and dark varieties. The total phenol and flavonoid content, which are the main active substances in tea, were also determined. Different types of tea were used to infer stable conclusions from studies, as different varieties, locations, processing conditions, and tea types have been reported to affect tea quality, bioactive components, and antioxidant capacity [22,23]. Generally, the total phenol and flavonoid content in green tea is significantly higher than in other teas, and its antioxidant index is also a key distinguishing feature. However, the antioxidant index and total phenolic and flavonoid contents of dark tea were relatively lower than those of other types of tea.

The antioxidant activity of tea leaves was determined using DPPH, FRAP, ABTS, and antioxidant indices of total reducing power. Tea bioactive compounds may interact through a variety of antioxidant mechanisms, including chelating metal ions, absorbing UV radiation, scavenging ROS, or non-free radical conversion and singlet oxygen inactivation. Tea showed different levels of antioxidant activity based on DPPH, FRAP, ABTS, and total reducing power (Figure 1). DPPH capacity is reflected as a decrease in absorbance that is proportional to the concentration and antioxidant activity of the tea extract [24,25]. FRAP assesses the reduction ability of tea leaves to Fe^3+^ and enables antioxidant reactions of tea leaves with Fe^2+^ complexes, reflecting the interaction and chelation ability of metal ions in living systems [26,27]. The ability to scavenge ABTS radicals reflects a reduction in superoxide radicals, a functional characteristic of living cells that inhibit lipid peroxidation [26,27]. These findings are supported by previous studies, which have shown that different types of tea have considerable antioxidant capacity [28,29]. The ability of tea to scour free radicals and ROS suggests that tea can be used as an antioxidant beverage or phytotherapy to treat cellular oxidative and inflammatory responses, especially those associated with HUA.

Tea samples (*n* = 101) exhibited significant differences in antioxidant capacity. In general, the antioxidant capacities of the teas were as follows: green > yellow > oolong > white > black > dark (high to low) (Table 1). Green and yellow tea showed the highest antioxidant capacity, with total phenolic contents of 158.58 ± 4.15 and 135.62 ± 4.18 (mg gallic acid equivalent/g tea) and total flavonoids contents of 122.24 ± 4.17 and 107.1 ± 6.23 (mg rutin equivalent/g tea), respectively. This may be related to the different fermentation processes, i.e., none and light. Among teas, green tea ranks the highest in terms of antioxidant capacity, which may be due to the lowest oxidation of tea leaves and the inactivation of PPO and POD enzymes during green tea production [28,29]. Catechins are the most important polyphenols in green tea. Their proportions are up to 25–35% of the leaves and they are responsible for the bitterness and astringency of the tea. Green tea contains the highest content of catechins. The catechins composition includes Epigallocatechin gallate (EGCG), Epicatechin (EC), Epigallocatechin (EGC), Epicatechin gallate (ECG), Catechin gallate (CG), Gallocatechin (GC), and Gallocatechin gallate (GCG), which present about 50–65% of the total catechins in green tea [30,31,32]. Partially fermented and lightly fermented oolong, yellow, and white tea showed comparable or similar antioxidant capacity to green tea. Several previous studies also reported that oolong tea and yellow tea contain high polyphenol contents, such as catechins (EGCG, EGC, GC, and ECG), flavonols and their glycosides, and bi-oactive catechin dimers that include theaflavin, thearubicin, and theafocin [33,34].

The antioxidant capacity of white tea is lower than that of green and yellow tea (Table 1), likely because of the simple processing steps. The tender shoots of white tea are harvested when most biological activities are being formed and degrading enzymes (polyphenol oxidase and peroxidase) remain active. In addition, polyphenols in white tea are degraded due to inactivating enzymes, enabling enzymatic and non-enzymatic degradation of the bioactive compounds of tea associated with antioxidant capacity to occur during the wilting process (prior to the drying stage) [3,35,36]. In addition, dark tea exhibits relatively low antioxidant values, which may be affected by the level and extent of fermentation during the production of dark tea. Correspondingly, the total phenols and flavonoids of dark tea and fully fermented black tea were also low. For example, the total phenols and flavonoids in dark tea were nearly two times lower than those in green tea. These findings are similar to previous work showing that antioxidant capacity decreases as the fermentation level of tea increases from green to black tea [37,38,39,40,41]. This study only determined the total content of phenolic and flavonoid compounds, and did not conduct qualitative or quantitative analysis of various components in tea (e.g., catechins, theaflavins, thearubigins). Therefore, mechanistic interpretations regarding specific metabolites in tea remain unverified.

Intake or supplementation of a diet containing potent antioxidants, such as vitamin C, reduces serum uric acid levels as well as the risk of HUA and associated comorbidities, including gouty arthritis [42]. In addition, uric acid acts as a potential antioxidant in plasma to scavenger ROS and protects the endothelial membrane from oxidation. However, HUA induces oxidative stress and endothelial dysfunction, leading to the occurrence of all-cause metabolic diseases, such as type 2 diabetes, cardiovascular disease, chronic kidney disease, NAFLD, and inflammatory diseases [43,44]. In addition, during UA formation, XOD hydrolyzates purines to produce nitric oxide (NO) from nitrile, whereas peroxides and hydrogen peroxide are also formed, contributing to cellular ROS elevation [42]. Therefore, XODi can be used to treat HUA. XOD inhibition decreased serum UA and reduced vascular oxidative stress. XODi drugs, including allopurinol, can be used to lower UA and treat gout and kidney stone disease, but they have some side effects [44,45]. Therefore, investigating the link between antioxidant and XODi activities in tea is important to mitigate HUA and maintain uric acid homeostasis.

The XODi activity of different tea leaves is shown in Figure 3. Black tea showed the numerically highest average XODi activity. The relatively high XOD inhibitory activity of black tea may hypothetically be attributed to oxidation-derived compounds, such as theaflavins and thearubigins, formed during full fermentation [31,46,47]. However, the XODi activity of green, yellow, and black tea was classified into one group according to Duncan’s multiple range test (*p* < 0.05). Green tea showed the highest TPC, TFC, and antioxidant capacity, while black tea exhibited the highest average XOD inhibitory activity. This clearly indicates that XOD inhibition activity does not simply depend on total phenol content or total antioxidant capacity, and may be related to specific functional components or their derivatives formed during tea processing. Dark tea exhibited some XODi activity, which is likely attributable to the addition of biological activities, such as emodin, to dark tea during microbial fermentation, resulting in XODi activity [48,49,50]. Correlation analysis showed that tea XODi activity was positively correlated with antioxidant activity, total phenols, and total flavonoids (Figure 5). Multivariate analysis by PCA revealed a good positive relationship between green tea type and the study indicators, with the highest principal component scores (Figure 6). The low XODi value of dark tea may be closely related to its unique processing technology and changes in chemical composition. During the fermentation of dark tea in a wood pile, polyphenols (especially catechins) are significantly reduced through long-term oxidation, leading to a decrease in antioxidant activity. In addition, microbial metabolism may degrade some amino acids during the fermentation in a wood pile, affecting their potential to participate in REDOX reactions. Green tea showed the highest TPC, TFC, and antioxidant capacity, while black tea exhibited the highest average XOD inhibitory activity. This clearly indicates that XOD inhibition activity does not simply depend on total phenol content or total antioxidant capacity, and may be related to specific functional components or their derivatives formed during tea processing.

The PCA model indicated some overlap in tea groups; therefore, the partial least squares discriminant analysis (PSL-DA) multivariate test could be performed. PLS-DA was performed and the tea samples were clearly classified from the multivariate dataset (Figure 7). Overall, PCA and PLS-DA models showed class separation in highly dispersed multivariate data, further revealing the classification of different tea species. Green tea was clearly separated from other types of tea, which scored high and were highly correlated with antioxidant activity and XODi activity (Figure 4 and Figure 7). A discrepancy was observed between the antioxidant capacity ranking (green > yellow > oolong > white > black > dark) and the XOD inhibitory activity ranking (black > green > yellow > white > dark > oolong). Although a positive correlation was found between them, their rankings were not completely consistent. This suggests that antioxidant capacity mainly relies on the total content of phenols and flavonoids, while XOD inhibitory activity may be more dependent on the specific structure of individual compounds rather than total content, which requires further verification. In combination with the experimental data, green tea was selected as the tea group with the best antioxidant capacity and XODi activity. The results of this study can be supported by the results of previous studies that reported that compounds with potent biological activity in green tea, such as catechins (EGCG and ECG) and phenolic acids, showed high antioxidant capacity [51,52,53].

## 5. Conclusions

In this study, the total phenols, total flavonoids, antioxidant capacity, and xanthine oxidase inhibitory activity of 101 tea samples (green, white, yellow, oolong, black and dark tea) were evaluated. Green, white, oolong, yellow, black, and dark tea exhibited varying antioxidant capacity. Xanthine oxidase inhibition activity was higher in black tea and lower in dark tea compared with that in the other types of tea. In terms of other antioxidant indexes, green and yellow tea had higher content, whereas dark tea had lower antioxidant indexes than that in other kinds of tea. Overall, green tea exhibited the strongest overall antioxidant profile with the highest contents of total phenols and total flavonoids, while black tea showed the highest mean XOD inhibitory activity, with no significant difference compared with green and yellow teas. Total phenol and flavonoid contents showed a significant positive association with antioxidant capacity and xanthine oxidase inhibitory activity in tea. Green tea has high total phenols, flavonoids, antioxidants, and xanthine oxidase in-hibitory activities, exhibiting potential to be developed into functional uric ac-id-lowering functions. Consumers should consider the observed antioxidant capacity and xanthine oxidase inhibitory activity when choosing green tea to maintain uric acid homeostasis and relieve HUA. In summary, this study provides valuable in vitro screening evidence for evaluating the antioxidant and xanthine oxidase inhibitory potentials of six major tea types. However, this study has certain limitations in terms of compound-specific analysis, sample origin, differences between extraction conditions and normal tea infusions, assay interference, and biological validation. Therefore, further in-depth chemical characterization targeting key phytochemicals including catechin monomers (EGCG, EC, EGC, ECG, CG, GC, GCG), theaflavins, thearubigins, flavonols and their glycosides, and phenolic acids using high-performance liquid chromatography (HPLC), ultra-high-performance liquid chromatography–tandem mass spectrometry (UPLC-MS/MS), nuclear magnetic resonance (NMR) spectroscopy, liquid chromatography–tandem mass spectrometry (LC-MS/MS), and other analytical methods combined with in vivo biological validation is indispensable to establish scientific and reliable functional or dietary recommendations.

## Figures and Tables

**Table 1:** Multiple comparison of studied variables in different tea groups.

Tea Types	XODi (%)	DPPH (mg Trolox Equivalents/g Tea)	FRAP (mg Trolox Equivalents/g Tea)	ABTS (mg Trolox Equivalents/g Tea)	TrdC (mg Trolox Equivalents/g Tea)	TPC (mg Gallic Acid Equivalents/g Tea)	TFC (mg Rutin Equivalents/g Tea)
Green tea (*n* = 26)	63.21 ± 1.63 ^a^	494.32 ± 12.41 ^a^	434.88 ± 13.2 ^a^	614.99 ± 21.87 ^a^	257.81 ± 8.56 ^a^	158.58 ± 4.15 ^a^	122.24 ± 4.17 ^a^
White tea (*n* = 32)	55.96 ± 1.23 ^ab^	390.83 ± 11.44 ^b^	256.13 ± 9.04 ^d^	345.74 ± 12.34 ^c^	156.49 ± 4.86 ^c^	102.98 ± 2.54 ^c^	88.32 ± 3.39 ^c^
Yellow tea (*n* = 16)	61.91 ± 1.84 ^a^	447.31 ± 13.22 ^a^	384.38 ± 11.61 ^b^	494.09 ± 20.06 ^b^	192.47 ± 10.86 ^b^	135.62 ± 4.18 ^b^	107.1 ± 6.23 ^b^
Oolong tea (*n* = 9)	45.69 ± 3.92 ^b^	392.36 ± 27.51 ^b^	316.45 ± 16.47 ^c^	493.43 ± 37.22 ^b^	153.82 ± 23.06 ^c^	117.99 ± 6.77 ^c^	81.79 ± 6.21 ^cd^
Black tea (*n* = 8)	63.95 ± 4.84 ^a^	295.49 ± 39.83 ^c^	215.93 ± 32.42 ^e^	334.82 ± 45.22 ^c^	127.77 ± 19.34 ^d^	98.89 ± 12.78 ^cd^	78.94 ± 10.86 ^cd^
Dark tea (*n* = 10)	48.15 ± 4.84 ^b^	256.15 ± 38.87 ^c^	187.07 ± 35.47 ^e^	247.04 ± 50.09 ^d^	97.76 ± 19.76 ^e^	75.36 ± 9.86 ^d^	62.95 ± 6.76 ^d^

Same superscript letter indicates no significant difference among tea samples in the studied variable column wise (*p* < 0.05). Values represent mean plus standard error. XODi: xanthine oxidase inhibitory; DPPH:1,1-diphenyl-2-picrylhydrazyl; FRAP: ferric ion reducing antioxidant power; ABTS: 2,2′-azino-bis(3-ethylbenzothiazoline-6-sulfonic acid; TrdC: total reducing capacity; TPC: Total phenol content; TFC: total flavonoid content.

## Data Availability

The original contributions presented in the study are included in the article/Supplementary Materials, further inquiries can be directed to the corresponding authors.

## Supplementary Materials

The following supporting information can be downloaded at: https://www.mdpi.com/article/10.3390/foods15101822/s1, Table S1: The origin of tea.

## Abbreviations

The following abbreviations are used in this manuscript:

DPPH	1,1-diphenyl-2-picrylhydrazyl
FRAP	Ferric ion reducing antioxidant power
ABTS	2,2′-azino-bis (3-ethylbenzothiazoline-6-sulfonic acid)
TrdC	Total reducing capacity
UA	Uric acid
TPC	Total phenol content
TFC	Total flavonoid content
XOD	Xanthine oxidase
XODi	Xanthine oxidase inhibitory
HUA	Hyperuricemia
PCA	Principal component analysis
PLS-DA	Partial least square discriminant analysis
ROS	Reactive oxygen species
CVDs	Cardiovascular diseases
NAFLDs	Non-alcoholic fatty liver diseases
H_2_O_2_	Hydrogen peroxide
•O_2_^−^	Superoxide ion
^1^O_2_^−^O_2_^−^	Singlet oxygen
OH	Hydroxyl radical
EGCG	Epigocatechin gallate
EC	Epicatechin
EGC	Epigallocatechin
ECG	Epicatechin gallate
CG	Catechin gallate
GC	Gallocatechin
GCG	Gallocatechin gallate
